# Artificial intelligence in medicine: mitigating risks and maximizing benefits via quality assurance, quality control, and acceptance testing

**DOI:** 10.1093/bjrai/ubae003

**Published:** 2024-03-04

**Authors:** Usman Mahmood, Amita Shukla-Dave, Heang-Ping Chan, Karen Drukker, Ravi K Samala, Quan Chen, Daniel Vergara, Hayit Greenspan, Nicholas Petrick, Berkman Sahiner, Zhimin Huo, Ronald M Summers, Kenny H Cha, Georgia Tourassi, Thomas M Deserno, Kevin T Grizzard, Janne J Näppi, Hiroyuki Yoshida, Daniele Regge, Richard Mazurchuk, Kenji Suzuki, Lia Morra, Henkjan Huisman, Samuel G Armato, Lubomir Hadjiiski

**Affiliations:** Department of Medical Physics, Memorial Sloan-Kettering Cancer Center, New York, NY, 10065, United States; Department of Medical Physics, Memorial Sloan-Kettering Cancer Center, New York, NY, 10065, United States; Department of Radiology, Memorial Sloan-Kettering Cancer Center, New York, NY, 10065, United States; Department of Radiology, University of Michigan, Ann Arbor, MI, 48109, United States; Department of Radiology, University of Chicago, Chicago, IL, 60637, United States; Office of Science and Engineering Laboratories, Center for Devices and Radiological Health, U.S. Food and Drug Administration, Silver Spring, MD, 20993, United States; Department of Radiation Oncology, Mayo Clinic Arizona, Phoenix, AZ, 85054, United States; Department of Radiology, University of Washington, Seattle, WA, 98195, United States; Biomedical Engineering and Imaging Institute, Department of Radiology, Icahn School of Medicine at Mt Sinai, New York, NY, 10029, United States; Office of Science and Engineering Laboratories, Center for Devices and Radiological Health, U.S. Food and Drug Administration, Silver Spring, MD, 20993, United States; Office of Science and Engineering Laboratories, Center for Devices and Radiological Health, U.S. Food and Drug Administration, Silver Spring, MD, 20993, United States; Tencent America, Palo Alto, CA, 94306, United States; Radiology and Imaging Sciences, National Institutes of Health Clinical Center, Bethesda, MD, 20892, United States; Office of Science and Engineering Laboratories, Center for Devices and Radiological Health, U.S. Food and Drug Administration, Silver Spring, MD, 20993, United States; Computing and Computational Sciences Directorate, Oak Ridge National Laboratory, Oak Ridge, TN, 37830, United States; Peter L. Reichertz Institute for Medical Informatics, TU Braunschweig and Hannover Medical School, Braunschweig, Niedersachsen, 38106, Germany; Department of Radiology and Biomedical Imaging, Yale University School of Medicine, New Haven, CT, 06510, United States; 3D Imaging Research, Department of Radiology, Massachusetts General Hospital and Harvard Medical School, Boston, MA, 02114, United States; 3D Imaging Research, Department of Radiology, Massachusetts General Hospital and Harvard Medical School, Boston, MA, 02114, United States; Radiology Unit, Candiolo Cancer Institute, FPO-IRCCS, Candiolo, 10060, Italy; Department of Translational Research and of New Surgical and Medical Technologies, University of Pisa, Pisa, 56126, Italy; Division of Cancer Prevention, National Cancer Institute, National Institutes of Health, Bethesda, MD, 20892, United States; Institute of Innovative Research, Tokyo Institute of Technology, Midori-ku, Yokohama, Kanagawa, 226-8503, Japan; Department of Control and Computer Engineering, Politecnico di Torino, Torino, Piemonte, 10129, Italy; Radboud Institute for Health Sciences, Radboud University Medical Center, Nijmegen, Gelderland, 6525 GA, Netherlands; Department of Radiology, University of Chicago, Chicago, IL, 60637, United States; Department of Radiology, University of Michigan, Ann Arbor, MI, 48109, United States

**Keywords:** artificial intelligence, radiology, machine learning, quality assurance, quality control, acceptance testing, deep learning

## Abstract

The adoption of artificial intelligence (AI) tools in medicine poses challenges to existing clinical workflows. This commentary discusses the necessity of context-specific quality assurance (QA), emphasizing the need for robust QA measures with quality control (QC) procedures that encompass (1) acceptance testing (AT) before clinical use, (2) continuous QC monitoring, and (3) adequate user training. The discussion also covers essential components of AT and QA, illustrated with real-world examples. We also highlight what we see as the shared responsibility of manufacturers or vendors, regulators, healthcare systems, medical physicists, and clinicians to enact appropriate testing and oversight to ensure a safe and equitable transformation of medicine through AI.

## Introduction

Artificial intelligence (AI) tools have the potential to revolutionize all aspects of medicine, from decision support in diagnosis to workflow management, drug discovery, and across the entire imaging chain in radiology. However, their integration into the clinical setting faces challenges, such as limited generalizability and fragility in real-world scenarios, that are exacerbated by a lack of transparency.^1–10^

Despite the promise of AI tools in medicine, the absence of standardized quality assurance (QA) protocols designed to evaluate performance in the local context to ensure patient and provider safety increases the risk of widespread errors and unintended consequences.^11^^,^^12^ For instance, the Epic sepsis model, a proprietary AI-driven system, was reported to have a substantial gap between its reported and local performance by independent auditors.^19^ Similarly, in 2017, Argentina's Salta province deployed an AI tool to identify adolescents at high risk of pregnancy; independent auditors found that the tool had inflated predictive accuracy because it had been trained and evaluated on nearly identical and biased datasets.^10^ Even AI tools that received regulatory clearance for clinical use may underperform when deployed in new clinical settings due to poor generalization or off-label use.^8^^,^^13^ These cases highlight the challenges faced by AI tools in medicine due to biases in development data (ie, training, validation, and test sets used by the developer to create the tool) and the potential distribution shifts in the characteristics of external, previously unused test sets or patient cases that reflect the local context.^10–12^^,^^14^ For the ethical and effective integration of AI tools into the clinical workflow, transparency from manufacturers about the development process and QA programs is necessary.^10^

Implementing AI tools into clinical practice is a shared responsibility between manufacturers and end-users^2^ that should mirror the QA programs required to install medical imaging devices.^15^ The programs should include comprehensive acceptance testing (AT) and continued, periodic quality control (QC) procedures. End-user training and a proper trial period with the local patient population should be required to ensure an understanding of the intended use and limitations of the AI tools before the AI recommendation may influence clinical decisions.^3^^,^^11^^,^^12^ The American Association of Physicists in Medicine (AAPM) Task Group (TG) 273 report provides a framework for AI tool testing and evaluation^11^ prior to clinical deployment. The pressing challenge is to develop rigorous QA procedures that maximize benefits, minimize risks, and are practical to implement in a clinical setting. This challenge instigated the formation of the multi-disciplinary AAPM group, TG 416, titled “Quality Assurance and User Training of CAD-AI Tools in Clinical Practice.” TG 416 aims to provide best practices for the QA of AI tools in medicine. The current commentary serves as an introductory discourse to TG 416, stressing the importance and function of QA in safeguarding patient care by ensuring the quality and safety of any AI tool used within the healthcare sector.

## Quality assurance—the act of responsibly ensuring the integration of AI tools in medicine

**Figure 1.**
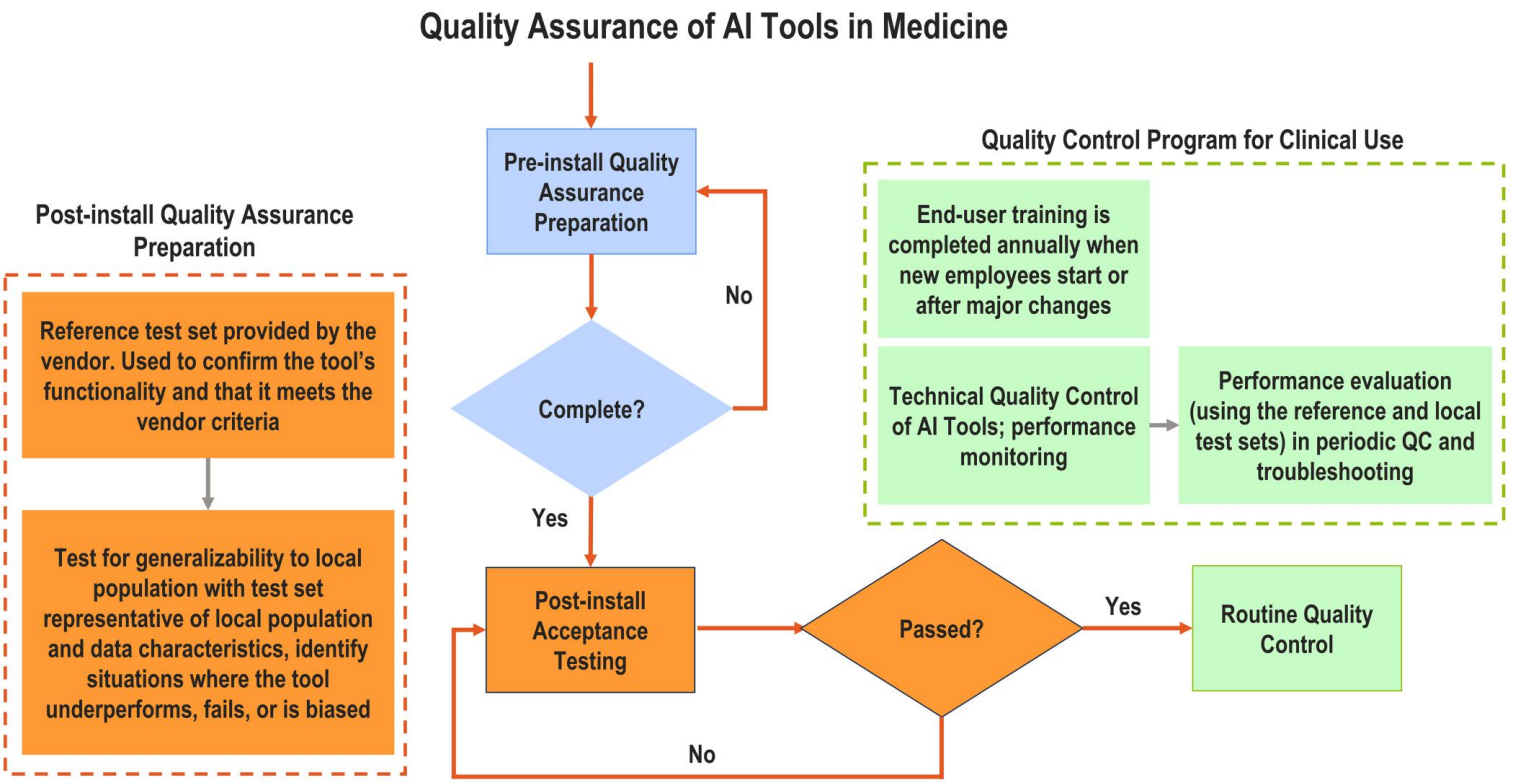
Flowchart illustrating the interconnected processes of quality assurance (QA), acceptance testing (AT), and quality control (QC) for AI tools in medical settings. The figure delineates the key steps emphasizing the cyclical nature of these processes for continuous improvement and patient safety. Some information that may be necessary for AT or the overall QA program must be obtained and reviewed before purchasing the AI tool. At installation of any AI tool, the necessary information must be provided to the team performing AT and ongoing QC procedures.

Quality control, a vital part of QA, consists of distinct technical procedures or checks for end-users to implement. The QA program encompasses initial AT and periodic QC procedures (Figure 1) that aim to identify, isolate, and resolve any issues before they impact patients.^3^^,^^11^^,^^12^ Given the technical complexities of AI algorithms, these guidelines should be practical and accessible to medical professionals who may not be AI experts. As specific procedures vary across AI tools, manufacturers should offer detailed guidelines on system setup, protocols, expected performance metrics for vendor-supplied reference datasets, and ongoing QC tests.^12^ They should also specify tolerance limits for both initial installation and future upgrades. Ideally, they should offer software tools to automatically track specific performance benchmarks over time. Additionally, user-friendly and efficient reporting tools for clinicians should be provided to document instances in which an AI tool provides unreasonable recommendations during routine use. Details on the development data, including demographic composition and intended use, should also be disclosed, so users can better understand the potential limitations of the tool in the local population.

Medical imaging has a longstanding commitment to quality and safety, upheld through stringent QA protocols. For example, mammography in the United States is governed by the Mammography Quality Standards Act (MQSA), which requires a comprehensive QA program consisting of initial AT, ongoing QC testing of the hardware and software, equipment maintenance, initial and continuous education, and peer-reviewed medical audits.^16^ The MQSA framework could serve as a valuable model for crafting QA guidelines for medical AI tools, targeting performance stability, user training standards, continuous education, and regular peer reviews.

One specific aspect of the MQSA framework that could be particularly beneficial for AI tools in medicine is the requirement for facilities to conduct regular audits, including peer reviews of diagnostic outcomes. Applying this principle to AI tools means establishing mechanisms for continuously evaluating the tools' performance in real-world clinical settings. For example, implementing systems to track specific safety and quality metrics is crucial in the context of AI tools used for diagnostic purposes. The accuracy of AI-generated diagnoses against confirmed clinical outcomes can be assessed, similar to how MQSA rules require facilities to compare mammographic findings with biopsy results annually. This process can confirm that AI tools are operating as intended. Moreover, continuous education, which is vital in MQSA for operators of mammography equipment, is equally essential for users of AI tools. Within medical imaging settings, QA is the core responsibility of medical physicists, who often interact with all teams installing new equipment. As such, the evaluation of AI tools in medicine could very well fall under the purview of the accreditation programs for physicists, interpreting physicians, and technologists.

Given the diverse applications of AI in medical imaging, each AI tool will require its own specific QA program. However, the general principle should be to assess each tool's functionality locally using well-curated, reference test sets with sufficient annotated cases for each subgroup in the local patient population. This approach involves evaluating the AI tool's performance across diverse patient subgroups that cover the local real-world patient population of interest, including subgroups that might be underrepresented in the initial training or pre-release test data. A carefully designed testing regime goes beyond mere accuracy metrics; it critically examines potential biases, sensitivity to specific anatomical variations, and the tool's adaptability to different clinical contexts. The increased scrutiny ensures that the AI tool operates equitably across a broader spectrum of patients, thereby building trust in its ability to generalize to the unique components of the local context. Additionally, the QA process should be tailored to the specific application, associated risks, and clinical environment in which it will be used.

In general, a QA program should include four steps:

1.  AT of newly installed tools, which is typically more rigorous than the ongoing routine QC tests.
2.  Determination of baseline performance.
3.  Ongoing monitoring of the tools to ensure early detection of any changes in performance.
4.  Periodic re-validation to verify performance after any changes to the workflow that could impact the AI tool output.

### Preparation for AI integration, transparency, and acceptance testing

AI tools, categorized as medical devices, are susceptible to subtle or pronounced failures that can negatively impact patient care and introduce liabilities. For example, Voter et al found reduced diagnostic accuracy in a commercially available AI tool on a local test dataset.^8^ In some cases, the reasons for the errors remained unknown. These findings stress the importance of context-specific evaluation of AI tools with locally curated reference test sets.

Ultimately, effective AT ensures seamless integration of the AI tool into local workflows without disrupting existing functionalities.^5^^,^^11^^,^^12^ It also verifies performance, outlines limitations, and flags potential biases. Table 1 offers an overview of the key elements involved in AT.

**Table 1.** General overview of the key considerations for acceptance testing and quality assurance of AI tools in medicine.

Stage	Description	Critical considerations	Potential stakeholders^a^
Preparation	Information review prior to installation: The vendors must provide instructions for use with detailed guidance on system installation, AT, acceptance criteria at installation and subsequent upgrades, proper user interface configuration, vendor-provided reference dataset, and the expected performance level of the AI tool along with tolerance limits. In-house teams ensure infrastructure compatibility, acquire representative local datasets, identify gaps, and establish test protocols and plans.	Considerations regarding the composition of training data, the target variable used for training, and the dataset size are necessary since increasingly complex AI models are at risk of overfitting.^11^ In addition, at this stage, factors related to the compatibility of models with local equipment and software environment, regulatory compliance, and stakeholder engagement should be understood. Performance metrics for efficacy and efficiency must be established.	Administrators, manufacturers or vendors, AT and IT teams. Patient representatives or ethic teams may be considered too
Implementation	Integrating the AI tool within the local setting, interoperability, cybersecurity, calibrating the system, and confirming functionality with a vendor-provided reference dataset.^1^	IT auditing processes^b^, system calibration, ensuring proper input data compatibility, verifying AI output and user interface functions, data privacy and security, vendor support.	AT and IT teams
Retrospective Evaluation	AI performance testing with local test sets. Baseline AT results are documented to enable comparisons. Performing additional failure mode analyses or case review audits.	Baseline metrics include obtaining quantitative and subjective measures from clinical users. In addition, identifying potentially unintended biases or unfairness using subgroups of patients, and performance metrics that capture ethical measures.^c^	Clinicians, AT and IT teams
Prospective Evaluation	Evaluation of the AI tool in a real-world clinical setting to gain experience or when retrospective test sets are not readily available. In general, this step should be completed after the tool is installed but before clinical use to ensure clinical decisions are not influenced.^12^	AI performance in clinical workflow is recorded and analyzed by clinicians and AT team, compared to follow-up clinical outcomes for sufficiently large number of cases. Procedures should be established to identify and address harmful or incorrect recommendations.	Clinicians, AT and IT teams, administrators, manufacturers or vendors
Ethical Considerations	Ensuring alignment with ethical standards, regulations, and best practices, including informed consent (if needed) and transparency.	Ethical guidelines considering the need for informed consent, transparency in algorithms, accountability mechanisms, and bias assessment.	Clinicians, regulators, patient advisory groups, manufacturers, administrators
User Training and Support at AT	Providing comprehensive training and ongoing support to end-users, including feedback mechanisms before the tool is deployed for routine clinical use.	User training should include hands-on experience observing AI performance in real-world cases, to understand its intended use and limitations, establish proper levels of trust/confidence, and avoid off-label use or misuse. This can be conducted during the prospective evaluation period.	Manufacturers or vendors, end-users
Risk Management	Identifying, assessing, and mitigating potential risks associated with the AI tool, including legal and clinical risks.	Identifying the risk of off-label use, inflated performance metrics,^10^ risk mitigation strategies, emergency protocols, liability considerations, and patient safety measures.	Clinicians, administrators, risk management team
End-to-end Workflow during Installation	Consideration of the entire workflow including training.	Comprehensive workflow consideration and optimization of all aspects of the AI tool usage.	AT and IT teams, manufacturers, or vendors

Abbreviations: AT = acceptance testing, IT = information technology.

The manufacturer creates the tool, establishing QA protocols, seeking regulatory approval, and offering product updates or technical support. A vendor may be responsible for distributing the tool and aiding with installation, user training, and support. The testing procedures required will depend on the tool, the risk it poses, and regulatory and manufacturer or vendor requirements.

a There may be more stakeholders or involvement than indicated, depending on resources at the local institution. The QA team generally includes clinicians, physicists, and technologists. Other technical personnel including AI domain experts, data scientists, statisticians, etc., may be involved if available and if needed.

b Integration of the device within the local setting, similar to the IT auditing processes established for cloud computing or cybersecurity, should be confirmed before testing the AI tool's functionality with vendor-supplied and locally acquired test datasets. Calibration is how well the predicted absolute risk corresponds to the true absolute risk.^11^ “Vendors” refers to groups that sell the AI tools, and “manufacturers” refers to those who develop the AI tools.

c AI tools must meet predefined performance and safety tolerance limits on retrospective and prospective case reviews before accepting for clinical use. Vendor-specified performance on the reference dataset and generalization performance on the local test sets should be documented as baseline results. Testing should also include assessing the tools’ performance on sub-groups, infrequent cases, and inputs with known artifacts that can reveal unintended biases or unfairness of the AI tool.

**Figure 2.**
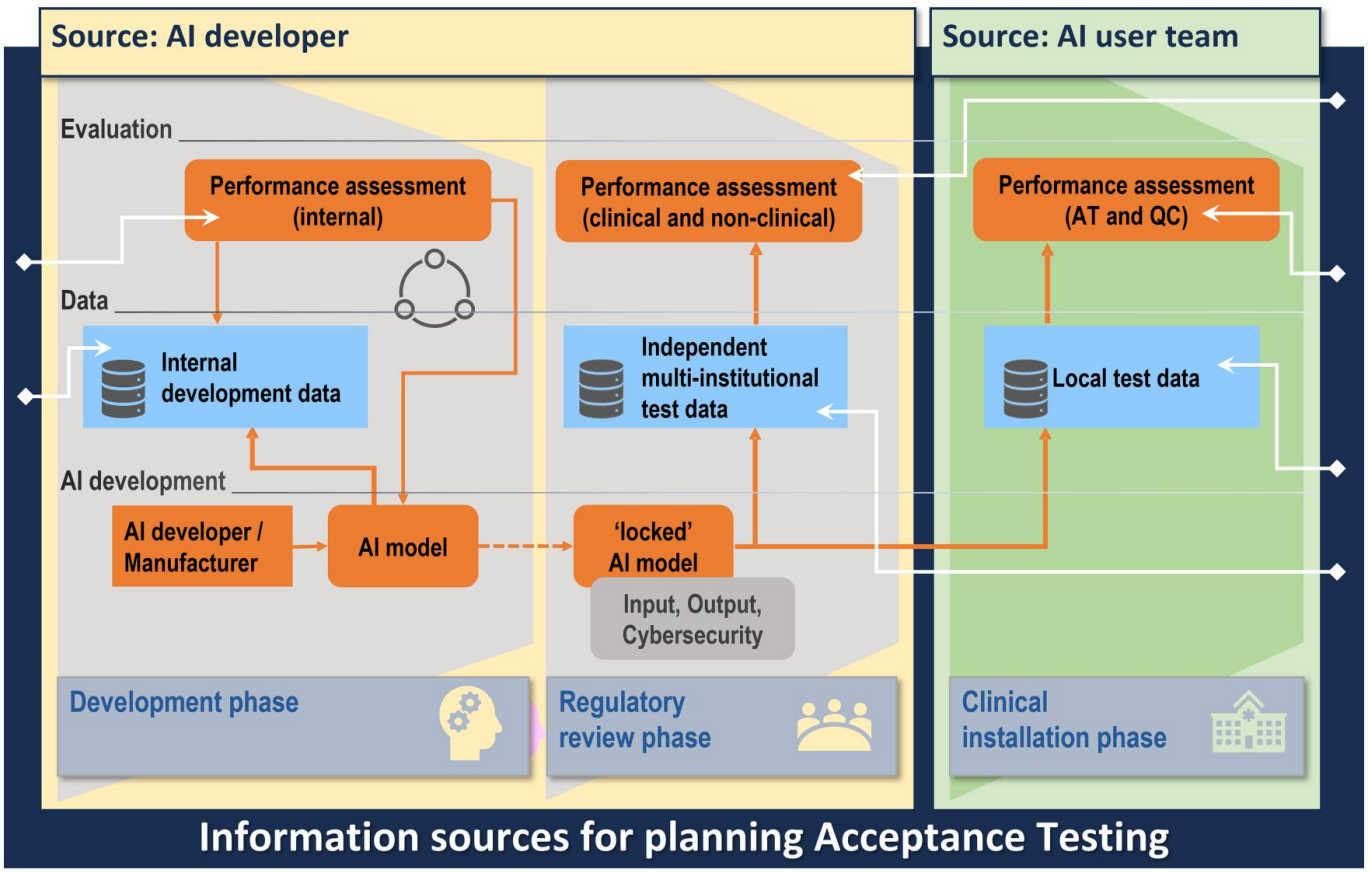
Overview of the different information sources involved in AI development, regulatory review, and clinical installation. Upon model completion (left), the locked model either undergoes regulatory review, additional retrospective or prospective multi-institutional validation (middle), or local clinical installation (right). Finally, before the tool is deployed for clinical use, testing with a site-specific, locally curated test set, the composition of which could be facilitated by vendor transparency, is essential.

Figure 2 outlines the range of information that the AI manufacturer should disclose during the initial purchasing or upgrade process and again to the teams performing the QA procedures, including demographics and characteristics (eg, sex, body mass index (BMI), age, race, type of equipment, and other confounding factors) of the development data. Such information is necessary to determine the relevance of the tool to a specific local patient population.^11^ Additional transparency about performance metrics and human factors, such as annotation procedures, is essential for building trust in the tool. Transparent communication from manufacturers or vendors is not only beneficial but essential for informed decision-making and safeguarding patient care.^11^

## Maintaining trust with ongoing, periodic quality control

While AT establishes baseline performance, ongoing QC is essential to monitor for any drifts in performance over time.^3^^,^^5^^,^^11^^,^^12^ The need for an ongoing QA program becomes even more critical if continuous-learning AI tools are introduced in the future. In addition, over time, the patient demographics or clinical workflow may change, shifting the local population characteristics (eg, patient age, BMI, conditions treated, imaging equipment or protocols). The evolved data may cause AI tools to drift from their initial performance. A routine QA program is indispensable for the timely detection of performance shifts in both the AI tool and the clinicians using it. The frequency of monitoring should be aligned with the risk that the tool poses (ie, higher risk should require more frequent audits). While not comprehensive, Table 2 gives a high-level overview of some factors that could lead to a malfunctioning AI tool.

**Table 2.** Clinical factors contributing to the malfunctioning of AI tools in medicine.

Category	Factors	Implications for ongoing QA
Shifts in Input Data	Changes in demographics, new hardware or software, change in image acquisition protocol, artifacts that impact input data quality, shifts in disease prevalence.^12^	Distribution or dataset shifts may cause AI tools to deviate from their baseline performance. The shifts may be anticipated due to planned changes or unexpected. They may also be isolated incidents due to off-label use (eg, adult tools used on pediatrics) or corrupted input (eg, poor image quality). Ongoing QA should include periodic review for distribution shifts and re-validation against the reference datasets.
Hardware Reliability	Hardware failure, updated hardware incompatibility, or general wear.	Physical component failures (eg, X-ray tube, detectors, sensors, etc.) affecting inputs or computational capabilities may impact performance and reliability of AI tools.^12^ QA procedures are contingent on the specific hardware configurations, the type of AI tool being used, and the unique operational environment in which it is deployed. QA should include regular hardware diagnostics and stress tests, especially for critical components, as instructed by the manufacturer or vendor.
Software Issues	Software bugs, version incompatibility, and security vulnerabilities in AI algorithms and supporting systems.	Ongoing QA may need to consider the interoperability of the AI tool with various medical data standards.^12^ Periodically assessing the tool’s compliance with evolving cybersecurity regulations is essential. QA should include regular security audits and penetration testing.
Data Integrity	Incomplete, incorrect, biased, or AI-derived input data.	Implement automatic QC check to monitor any drift of AI performance over time. If drift occurs, identify whether input data integrity is the cause.

### Multifaceted approach to QA

Given the multiple factors affecting human and AI performance, a comprehensive QA strategy is essential.^11^^,^^12^ This strategy could include tests on hardware, software, or AI system inputs at various intervals, eg, daily, monthly, or semi-annually. Manufacturers should identify local workflow elements that could affect AI output and offer monitoring tools. Additional QA procedures may be designed according to end-user feedback. In addition to manufacturer guidance, test frequency should consider the risk level, regulations (if they exist), and operational experience. Furthermore, frequent testing may be warranted for high-risk tools after significant changes in clinical workflow, technical updates, or unusual errors noticed by clinical users. The goal is to balance patient safety with operational efficiency.

**Figure 3.**
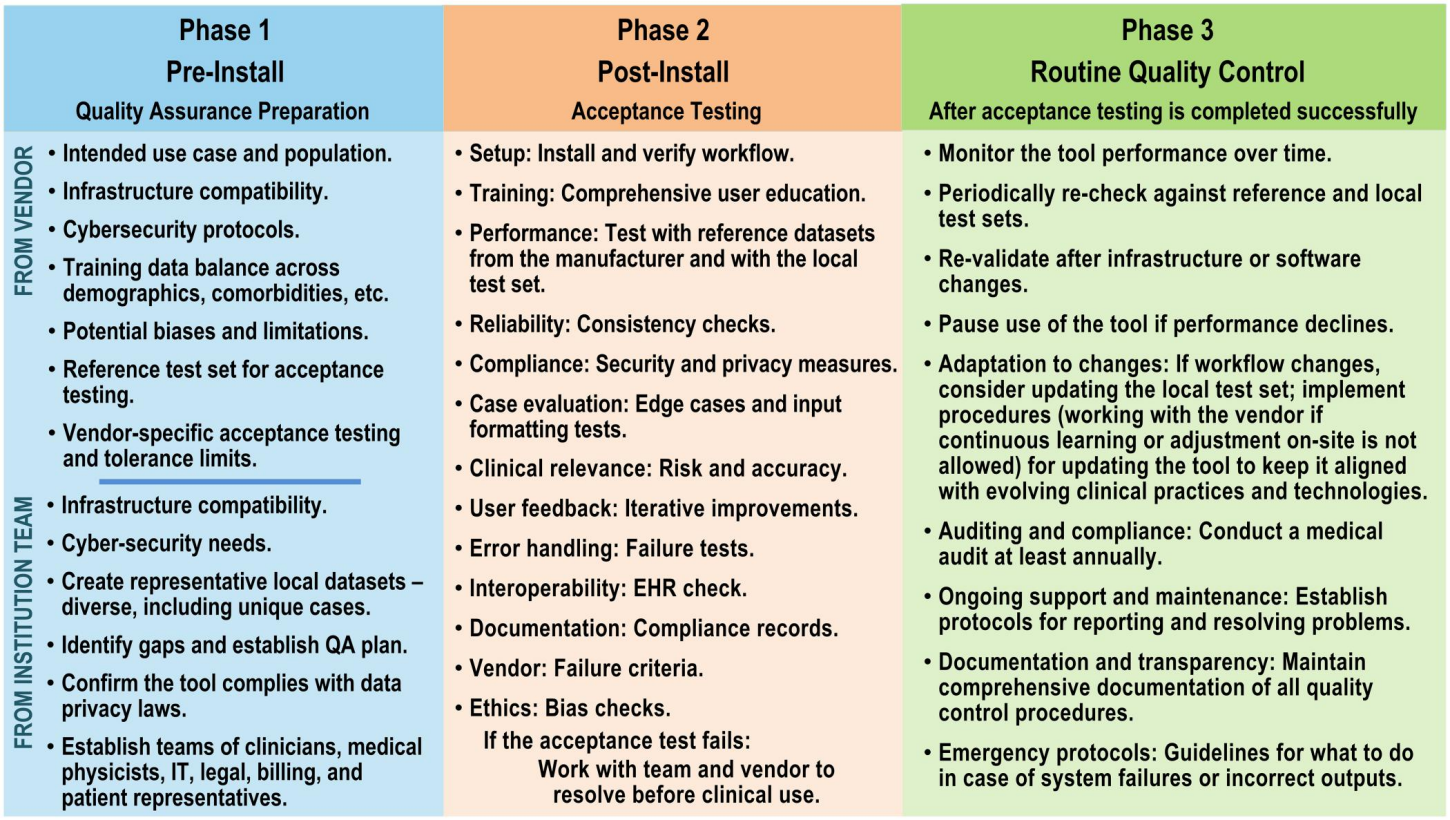
Example considerations for quality assurance (QA) in the 3 phases of AI tool integration into the clinical workflow: pre-install QA preparation, post-install acceptance testing, and routine quality control.

High-risk AI tools, such as those involved in triage or medical diagnoses, require rigorous annual assessment. The evaluations should scrutinize the tool for fairness, potential biases, and error rates. In addition, the clinicians' interactions with the AI tool should be reviewed to identify potential issues such as automation bias. The annual re-validation process may involve repeating AT procedures with the vendor-provided and locally curated reference test sets to identify any deviations from baseline performance. If changes in clinical workflow, patient demographics, imaging equipment or software upgrades, or other factors that could influence the AI tool occur, re-validation must be considered. In such cases, compiling an updated local reference test set that reflects the changes would also be advisable. Moreover, a peer-review mechanism is essential to identify performance shifts. Figure 3 elaborates on the components of a comprehensive QA program.

### Example QA workflow

AI-based auto-segmentation in radiation therapy^17^ can be used as an example for a general QA workflow for the clinical application of AI.

Step 1: Following the QA considerations in Table 1 and Figures 1-3, the first step requires forming a multidisciplinary team, identifying and justifying the clinical need, and reviewing available performance data from the development and regulatory approval phase.^18^ The local requirements of the AI tool must be identified including the business needs, the target use case and variables, anticipated equipment the tool will interface with, details about the local patient population,^12^ anticipated patient volumes, general purchase conditions, generic technical specifications of the AI tool, a request for acceptance testing and routine QA protocols, and a request for expected performance levels and metrics to assess the AI tool.^19^ The collected information should be documented in a detailed QA manual that describes each test, metrics used to assess performance, and the acceptability criteria.

Step 2: Post-installation, the successful integration of the AI tool within the local clinic is confirmed by IT, cyber-security, and other relevant parties as deemed necessary by the vendor and local site. AT, then, is conducted using vendor-supplied reference and locally curated test sets (Table 1 and Figures 1-3). The entire imaging chain or end-to-end workflow should be evaluated before the tool is allowed to influence clinical decision-making. A local test set should represent a diverse range of patient cases and include pixel-wise annotations by experts; patient cases in the local patient archive with clinically verified manual contours for treatment planning can be retrieved and de-identified for this purpose. A beta trial period with prospective evaluations could be conducted if a local test set is unavailable, as described in Table 1. For auto-segmentation, prospective testing requires comparing AI tool segmentation results with manual contours drawn by experts. A vendor-supplied tracking program could help quantify performance discrepancies and log cases or instances where the tool underperformed. The AI tool may be rejected if the performance fails the acceptance criteria. The results of the acceptance testing must be documented for future reference.

Step 3: Minor errors may be corrected, but significant inaccuracies requiring manual organ delineation from scratch necessitate deeper investigation, including vendor involvement. Subsequent routine QA testing of the tool should occur at intervals recommended by the vendor, regulatory bodies, or the QA team. Routine QA should encompass technical QC checks for tool reproducibility using the same local test set at acceptance testing, performance monitoring of the tools and human users, and annual training. Medical audits comparing outputs against ground truths and annual peer reviews against expert clinician segmentation are recommended.

## User training

Building on the essential roles of AT and QC, user training is a critical element for successfully integrating AI tools into healthcare. To encourage adoption and minimize risks, the end-users must understand the tool’s intended use, capabilities, limitations, and ethical implications. Such training should be both comprehensive and tailored to meet the unique requirements and protocols of each clinical site.^10–12^

In addition to application-specific instructions, training modules should include information on the correct usage of the AI tool, underlying assumptions, legal framework, and case studies illustrating both successful and unsuccessful applications. This multifaceted approach aids in understanding the tool's strengths and limitations. Crucially, user training should commence before the AI tool starts influencing clinical decisions and should be periodically updated throughout the AI tool's operational life. Continuous education should include peer-reviewed audits and equip clinicians to effectively communicate the role and impact of AI tools in patient care. Furthermore, settings where AI outputs guide downstream decisions warrant additional discipline-specific training. For example, auto-segmentation for radiotherapy planning requires robust education across dosimetrists, physicists, physicians, and oncologists interacting with the contours.^17^ Comprehensive training that includes all users empowers the local teams to effectively scrutinize AI outputs during treatment planning, identify deviations, and account for limitations.

## Conclusion

In summary, the ethical and effective deployment of AI in healthcare is substantially enhanced by rigorous QA protocols, transparent vendor practices, and a commitment to ongoing monitoring and adaptation. Through continuous monitoring and rigorous testing, QA ensures that medical AI tools remain reliable and effective across varied patient demographics and clinical scenarios. Rigorous testing procedures enhance their trustworthiness among clinicians and patients and support the broader goal of ensuring that AI tools can be effectively generalized to different settings. Integrating robust QA programs creates a more resilient healthcare system equipped to harness the benefits of AI while minimizing risks. These elements collectively contribute to making AI a more reliable, safe, and equitable tool in medicine, enabling healthcare providers to build trust and prevent harm while adapting to the evolving landscape of AI.

## Funding

K.D. was supported by MIDRC (The Medical Imaging and Data Resource Center) through the National Institute of Biomedical Imaging and Bioengineering (NIBIB) of the National Institutes of Health under contract 75N92020D00021. H.-P.C. was supported by the National Institutes of Health Award Number R01 CA214981. L.H. was supported by the National Institutes of Health Award Number U01-CA232931. R.M.S. was supported by the Intramural Research Program of the National Institutes of Health Clinical Center. J.J.N. was supported by National Institutes of Health (NIH) Grant Numbers R01CA212382, R01HL164697, and the Interim Support Funding of the Massachusetts General Hospital Executive Committee on Research (ECOR).
